# Assessment of genetic diversity and differentiation among four indigenous Turkish sheep breeds using microsatellites

**DOI:** 10.5194/aab-63-165-2020

**Published:** 2020-06-11

**Authors:** Bahar Argun Karsli, Eymen Demir, Huseyin Goktug Fidan, Taki Karsli

**Affiliations:** Department of Animal Science, Faculty of Agriculture, Akdeniz University, Antalya, 07058, Turkey

## Abstract

Conservation and breeding programmes of livestock species depend on determination of genetic diversity. Today in livestock species, microsatellite markers are commonly used to reveal population structure and genetic diversity in both breeds and varieties. In this study, population structure, genetic diversity, and differentiation among four native Turkish sheep breeds including Güney Karaman, Kangal, Norduz, and Karakas were assessed by using 21 microsatellite loci. By genotyping 120 individuals belonging to four sheep breeds, a total of 275 different alleles, 37 of which were private alleles, were observed across all loci. The mean number of alleles per breed ranged from 7.28 (Güney Karaman) to 8.09 (Karakas), while allelic richness ranged from 7.22 (Güney Karaman) to 7.87 (Karakas). Mean observed heterozygosity varied from 0.60 (Kangal) to 0.66 (Norduz and Karakas). The lowest pairwise $F_{\mathrm{ST}}$ value (0.084) was between Kangal and Karakas populations, while the highest pairwise $F_{\mathrm{ST}}$ value (0.142) was between Norduz and Karakas populations. Polymorphic information content (PIC) values, ranging from 0.71 (ETH10) to 0.91 (OarFCB304), were highly polymorphic (PIC > 0.5) and informative in studied populations. In the present study, the results of phylogenetic analysis were of importance, since all studied populations have been accepted as Akkaraman varieties till today. However, factorial correspondence and structure analysis, pairwise $F_{\mathrm{ST}}$ values, and an unweighted pair group method with arithmetic mean analysis (UPGMA) dendrogram revealed that Güney Karaman and Norduz populations have became genetically different from the Akkaraman breed due being raised in different parts of Turkey under different climatic conditions together with their breeding practices. Therefore, we recommend that more comprehensive molecular studies should be conducted to clarify genetic differentiation of Akkaraman sheep varieties.

## 1 Introduction

Sheep's milk and meat are important foodstuffs for the feeding of society. Sheep raising supports breeders' incomes and the economy of Turkey with nearly 34 million sheep (TUIK, 2019). A large part of the sheep population raised in Turkey is represented by native breeds. Although native sheep breeds have low milk and meat yields, they are resistant to temperature changes and diseases of raised regions (Soysal et al., 2005). Sheep raising has always been a part of cultural values of Turkey through its history. In rural areas, sheep rearing not only makes a contribution to breeders' incomes but also is a lifestyle known as nomadic sheep breeding. Furthermore, Turkey makes a contribution to world animal genetic resources with nearly 20 defined native sheep breeds (Ertugrul et al., 2009).

Mainly distributed in Middle Anatolia and nearby places, Akkaraman is the most raised breed with an approximately 40 % proportion among Turkish native sheep breeds (Karaca et al., 2003; Ertugrul et al., 2009). There are different varieties of the Akkaraman breed such as Güney Karaman (GKR), Norduz (NRD), Karakas (KRK), and Kangal (KNG) adapted to different regions (Karaca et al., 2003; Karsli et al., 2011). GKR, also known as black sheep, is reared in the Mediterranean region (especially in Antalya, Mersin, and Hatay provinces) in a nomadic breeding system (Karsli et al., 2011). The NRD variety is raised in Van Province, whereas KRK sheep are raised in Eastern Anatolia including Diyarbakır, Van, Bitlis, and Hakkâri provinces (Bingol and Aygun, 2014). KNG, raised extensively in Sivas and Malatya provinces, has the largest body size among variety of the Akkaraman breed (Kurar et al., 2012; Karsli et al., 2011). Since KNG sheep have been derived from the Akkaraman breed by selection in the recent past, they show similar phenotypic traits as Akkaraman sheep. On the contrary, GKR, NRD, and KRK sheep have differed from Akkaraman distinctively in terms of phenotypic traits due to rearing in different regions together with diverse adaptation processes for a long time.

It has been reported that GKR, NRD, and KRK breeds are in danger of extinction (Ertugrul et al., 2009). While GKR has been included in a conservation programme at Bahri Dagdas International Agricultural Research Institute authorized by the Ministry of Agriculture and Forestry, General Directorate of Agricultural Research and Policies, NRD and KRK breeds have been under a conservation programme handled by breeders (Ertugrul et al., 2009).

Genetic diversity shaped by evolution and climate changes is crucial for the survival of populations in different regions (Kumar et al., 2006). Sheep populations raised near the domestication centre have higher genetic diversity which decreases with increasing geographic distance (Kijas et al., 2009). There is strong evidence that the domestication centre of sheep is the Fertile Crescent including southeastern and Eastern Anatolia of Turkey (Alberto et al., 2018). Sheep were spread out from the Fertile Crescent to other regions of the world. Today, sheep are raised in almost all regions of the world including both desert and tropical regions due to their extraordinary adaptability (Amills et al., 2017).

Genetic diversity studies are the first step of conservation and breeding programmes. Many molecular markers such as RAPD (Kunene et al., 2009), AFLP (De Marchi et al., 2006), PCR-RFLP (Kucharski et al., 2019), and mtDNA (Kirikci et al., 2018) have been applied to detect genetic diversity in livestock. Today, SSRs (simple sequence repeats (Demir and Balcioğlu, 2019) and SNPs (single nucleotide polymorphisms) (Ilori et al., 2018) and NGS (next-generation sequencing) (Kijas et al., 2009) are preferred to reveal genetic diversity among and within different livestock breeds.

SSR markers are commonly used to reveal genetic diversity because they are abundant, distributed through genome randomly, easy to access and apply, highly polymorphic, and show codominant inheritance. SSR markers were used to detect genetic diversity or parentage test in different livestock including cattle (Demir and Balcioğlu, 2019), sheep (Ocampo et al., 2016), goat (Jawasreh et al., 2018), pigs (Szmatoła et al., 2016), horses (Semik and Za̧bek, 2013) chickens (Karsli and Balcioğlu, 2019), rabbit (Abdel-Kayf et. al., 2016), ducks (Hariyono et al., 2019), and donkeys (Han et al., 2017).

Despite the huge phenotypic variations among GKR, NRD, and KRK sheep, they are known as varieties of the Akkaraman sheep breed. Unfortunately, genetic studies focusing on population structure and phylogenetic relationship among these varieties are scarce. In addition, there are not enough studies revealing genetic diversity in NRD and GKR whose population sizes have decreased in the last few decades. Hence, this study aimed to determine genetic diversity and phylogenetic relationship among KNG, GKR, NRD, and KRK populations by using 21 SSR markers.

## 2 Methods

### 2.1 Collection of blood samples and genomic DNA extraction

**Figure 1:**
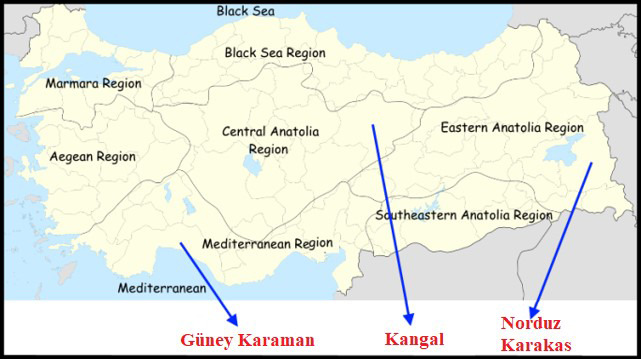
Geographical raising regions of four Turkish native sheep populations. Note: the base map of Turkey was downloaded from the web (https://tr.wikipedia.org/wiki/Dosya:Turkey_region_map_with_province_borders.svg, last access: 9 June 2020).

In this study, a total of 120 sheep (from both sexes) were chosen from four sheep breeds raised in different regions in Anatolia (Fig. 1). Samples belonging to KNG (n=30) sheep were collected from Sivas Province; NRD (n=30) and KRK (n=30) samples were collected from Van Province, and GKR (n=30) samples were collected from Antalya Province. Samples of each sheep breed were collected from at least three different farms between 2016 and 2018. No ethical approval was necessary for the present study, since blood samples were collected during routine veterinary visits. Genomic DNA was extracted from blood samples using salting-out method reported by Miller et al. (1988). DNA quality and quantity were determined using agarose gel (1 %) and spectrophotometer (NanoDrop-SD 1000). DNA concentration was adjusted to 50 ng µL for PCR process after DNA extraction.

#### 2.1.1 Microsatellite genotyping

In the present study, a total of 21 microsatellite loci, 14 of which are recommended by FAO (2011) to determine genetic diversity in sheep, were used. PCR amplifications were carried out in a total volume of 25 µL PCR mixture. PCR mixture consisted of 2–2.5 µL genomic DNA (50 ng µL${}^{-1}$), 1.2 µL HQ buffer (Geneall), 2 µL dNTPs (2.5 mM µL${}^{-1}$), 0.25 µL of each primer (10 pmol µL${}^{-1}$), 0.4 µL (2.5 U µL) Taq polymerase (Geneall), and distilled deionized water. PCR reaction conditions were performed as follows: initial denaturation at 95 ${}^{\circ }$C for 5 min, followed by 30 cycles of denaturation at 94 ${}^{\circ }$C for 45 s, annealing (at 50–60 ${}^{\circ }$C for different loci) for 45 s, extension at 72 ${}^{\circ }$C for 45 s, and final extension at 72 ${}^{\circ }$C for 5 min.

In this study, 96 automated capillary electrophoresis systems (Advanced Analytical Technologies, Iowa, USA) were used for fragment analysis. The capillary conditioning solution, inlet buffer, separation gel, and 35–500 bp marker were prepared according to the user manual provided by the manufacturer. After capillary electrophoresis separation, the raw data were recorded, and band sizes were calculated using PROSize^®^ 2.0 version 1.3.1.1 (Advanced Analytical Technologies, Iowa, USA).

#### 2.1.2 Statistical analysis

Number of private alleles (PA) and allele size range (ASR) was determined by using the Convert version 1.3.1 (Glaubitz, 2004) program. Convert version 1.3.1 (Glaubitz, 2004) was also used to convert data to other program's file formats. Number of alleles ($N_{a}$), number of effective alleles ($N_{e}$), observed ($H_{O}$) and expected heterozygosity ($H_{E}$) values, and genetic distance values among four sheep populations were calculated using the POPGENE version 1.31 (Yeh et al., 1997) software package. The Ml-Nullfreq program (Kalinowski and Taper, 2006) was used to calculate null allele frequencies for each locus in four sheep populations. Polymorphic information content (PIC) values were calculated with Excel Microsatellite Toolkit ver. 3.1 (Park, 2001); genetic differences (pairwise $F_{\mathrm{ST}}$) between the populations were determined by using Arlequin software ver. 3.1 (Excoffier et al., 2005). Inbreeding coefficient ($F_{\mathrm{IS}}$) and allelic richness values were obtained with FSTAT software v.1.2 (Goudet, 1995). The significance of $F_{\mathrm{IS}}$ values for populations was tested by using Arlequin software ver. 3.1 (Excoffier et al., 2005).

The phylogenetic relationship among studied sheep populations were determined according to unweighted pair group method with arithmetic mean analysis (UPGMA) dendrogram constructed on basis of Nei's standard genetic distance (Nei, 1987). The UPGMA dendrogram was obtained by the POPGENE version 1.31 (Yeh et al., 1997) program. In addition, factorial correspondence analysis (FCA) and Bayesian approach based on STRUCTURE clustering analysis were carried out. GENETIX ver. 4.05 (Belkhir et al., 2004) software was used for FCA analysis. Bayesian model-based clustering was constructed by using Structure software (Pritchard et al., 2000). One hundred independent runs were carried out for different genetic clusters (K is from 2 to 4; K is the number of clusters,) with a burn-in period of 100 000 iterations and a total of 500 000 Markov chain Monte Carlo (MCMC) under an admixture model and correlated allele frequencies. Structure Harvester (Earl and von Hold, 2012) was used to determine the most probable K value, by the calculation of the ΔK statistics as described by Evanno et al. (2005). The CLUMPAK software was used to visualize the Structure outputs (Kopelman et al., 2015).

## 3 Results

### 3.1 Genetic diversity among sheep populations

In this study, all microsatellite loci were polymorphic. Basic genetic diversity parameters and PIC values calculated for 21 microsatellite loci (Table 1). In four sheep populations, mean number of alleles and effective alleles were 13.10 and 7.78, respectively, across 21 microsatellite loci. The lowest (6.03) and the highest (13.74) allelic richness (AR) values were detected in ETH10 and CSRD247, respectively. PIC value ranged from 0.71 (ETH10) to 0.91 (OarFCB304) with a mean of 0.84.

**Table 1:** Descriptive statistics for genetic diversity over 21 microsatellite loci in four local Turkish sheep populations.

Locus	N	ASR	$N_{a}$	$N_{e}$	AR	PIC	F(Null)	Locus	N	ASR	$N_{a}$	$N_{e}$	AR	PIC	F(Null)
OarJMP58	112	129–163	15	8.11	12.40	0.87	0.13	MAF214	118	172–224	15	7.22	11.41	0.85	0.05
SPS115	114	232–256	12	7.66	10.98	0.86	0.11	MAF65	113	118–134	9	5.77	7.37	0.80	0.04
MCM527	114	153–175	12	6.81	10.86	0.84	0.15	BM1824	112	158–190	13	7.21	9.98	0.85	0.18
HSC	114	265–289	13	9.73	11.78	0.89	0.09	SRCRSP9	118	109–125	9	6.79	8.28	0.84	0.05
BM8125	113	114–130	9	7.17	8.75	0.84	0.09	CSSM66	118	156–212	17	10.35	13.29	0.90	0.13
ILTS28	114	144–176	17	8.13	12.66	0.87	0.04	SRCRSP1	114	122–142	10	4.52	7.09	0.75	0.05
OarJMP29	108	130–150	10	4.47	8.12	0.75	0.26	BM1818	115	230–280	18	10.22	13.62	0.89	0.00
ILTS11	113	256–292	14	10.38	12.24	0.90	0.06	MAF70	115	144–156	18	9.24	13.39	0.88	0.15
ETH10	120	200–212	7	4.02	6.03	0.71	0.25	SRCRSP5	115	144–156	7	5.65	6.87	0.80	0.18
OarFCB304	110	150–188	18	11.41	13.73	0.91	0.04	D5S1	105	180–216	15	7.92	10.53	0.86	0.18
CSRD247	118	211–247	17	10.58	13.74	0.90	0.12	Mean			13.10	7.78		0.84	0.11

N: total number of samples used at each locus; ASR: allele size range. $N_{a}$: number of alleles at each locus; $N_{e}$: effective number of alleles at each locus; AR: allelic richness; PIC: polymorphic information content; F(Null): null allele frequency.

Descriptive statistics together with private alleles for sheep populations are given in Table 2. The mean number of alleles per population ranged from 7.28 (GKR) to 8.09 (KRK), while AR ranged from 7.22 (GKR) to 7.87 (KRK). Mean observed heterozygosity varied from 0.60 (KNG) to 0.66 (NRD and KRK). Private alleles, another parameter of genetic differentiation, ranged from 7 (KRK) to 12 (KNG).

**Table 2:** Descriptive statistics for genetic diversity of each sheep population over 21 loci.

	$M_{N_{a}}$ ± S	$M_{N_{e}}$ ± SD	AR	$H_{O}$ ± SD	$H_{E}$ ± SD	PA	PIC	$F_{\mathrm{IS}}$
GKR	7.28 ± 2.30	4.86 ± 1.37	7.22	0.61 ± 0.22	0.78 ± 0.07	9	0.74	0.236${}^{**}$
NRD	7.62 ± 2.01	5.08 ± 1.60	7.47	0.66 ± 0.17	0.79 ± 0.08	9	0.74	0.170${}^{*}$
KNG	7.90 ± 2.07	5.45 ± 1.66	7.73	0.60 ± 0.21	0.81 ± 0.07	12	0.77	0.260${}^{**}$
KRK	8.09 ± 2.07	4.69 ± 1.38	7.87	0.66 ± 0.19	0.78 ± 0.06	7	0.75	0.155${}^{*}$
Mean	7.72 ± 0.35	5.02 ± 0.33	7.57	0.63 ± 0.03	0.79 ± 0.01	9.25	0.75	0.205

GKR: Güney Karaman; NRD: Norduz; KNG: Kangal; KRK: Karakas. $M_{N_{a}}$: mean number of alleles per breed; $M_{N_{e}}$: mean number of effective alleles per breed; AR: allelic richness per breed; $H_{O}$: average observed heterozygosity per breed; $H_{E}$: average expected heterozygosity per breed; $F_{\mathrm{IS}}$: Inbreeding coefficient; PA: number of private alleles; frequency higher than >10 %; SD: standard deviation. Significantly different from zero: ${}^{**}$ p<0.01; ${}^{*}$ p<0.05. PIC: the average polymorphic information content. Inbreeding coefficient value ranged from 0.155 (KRK) to 0.260 (KNG) with a mean of 0.205.

### 3.2 Genetic differentiation and phylogenetic analysis among populations

Pairwise $F_{\mathrm{ST}}$ values based on 21 microsatellite loci are given in Table 3 for each sheep population.

**Table 3:** Pairwise $F_{\mathrm{ST}}$ values among the studied four sheep breeds.

	GKR	NRD	KNG	KRK
GKR	–			
NRD	0.126${}^{*}$	–		
KNG	0.102${}^{*}$	0.092${}^{*}$	–	
KRK	0.100${}^{*}$	0.142*	0.084${}^{*}$	–

GKR: Güney Karaman; NRD: Norduz; KNG: Kangal; KRK: Karakas. ${}^{*}$ p<0.01.

The lowest pairwise $F_{\mathrm{ST}}$ value (0.084) was between KNG and KRK populations, while the highest pairwise $F_{\mathrm{ST}}$ value (0.142) was between NRD and KRK populations.

Nei's genetic distance values in studied populations are given in Table 4. The lowest genetic distance (0.088) was between KNG and KRK populations, while the highest genetic distance (0.153) was between KRK and NRD populations.

**Table 4:** Nei's (1987) genetic distance values among populations.

	GKR	NRD	KNG	KRK
GKR	–			
NRD	0.135	–		
KNG	0.107	0.097	–	
KRK	0.106	0.153	0.088	–

GKR: Güney Karaman; NRD: Norduz; KNG: Kangal; KRK: Karakas.

**Figure 2:**
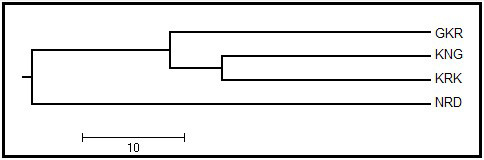
UPGMA dendrogram among four native sheep populations based on Nei's genetic distance values. GKR: Güney Karaman; NRD: Norduz; KNG: Kangal; KRK: Karakas.

A UPGMA dendrogram based on Nei's genetic distance values is given in Fig. 2 for studied populations. The UPGMA dendrogram classified studied populations in two branches in which GKR, KNG, and KRK constituted the first branch, while NRD population clustered separately in the other branch.

**Figure 3:**
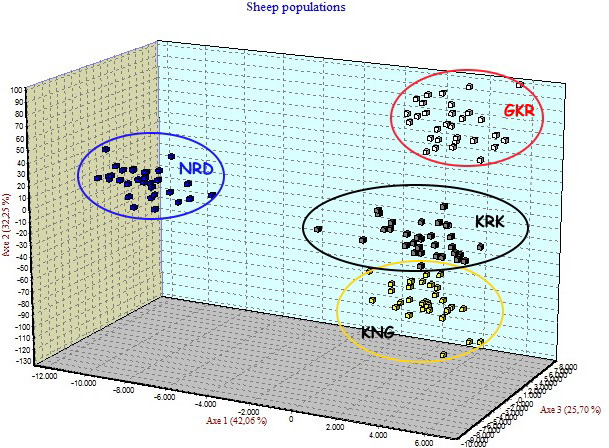
Factorial correspondence analysis among four sheep breeds. GKR: Güney Karaman; NRD: Norduz; KNG: Kangal; KRK: Karakas.

Four native Turkish sheep populations were located in three-dimensional space by FCA analysis given in Fig. 3. The FCA analysis separated the NRD and GKR populations very clearly, while KRK and KNG clustered close together.

**Figure 4:**
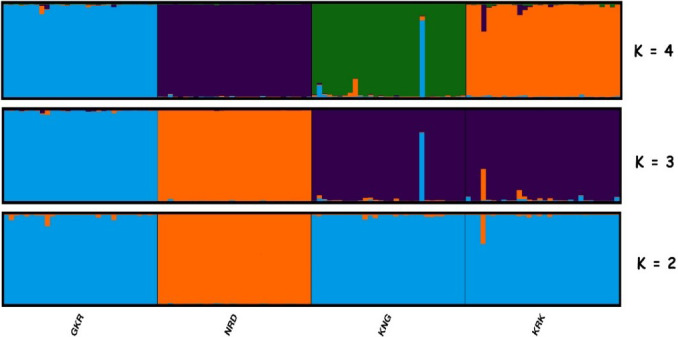
Structure cluster analysis of the studied individuals. The highest ΔK value was obtained at K=3. GKR: Güney Karaman; NRD: Norduz; KNG: Kangal; KRK: Karakas.

Structure analysis, another clustering method, revealed that GKR, KNG, and KRK populations clustered together, while NRD clustered separately at K=2 (Fig. 4). At K=3, determined by structure harvester to be most likely number of clusters, KNG and KRK populations clustered together, whereas NRD and GKR showed very clear separate clusters. These findings imply that although KNG and KRK populations may be typical varieties of the Akkaraman sheep breed, GKR and NRD populations, which showed clear differentiation in structure analysis, may be considered to be genetically different. In addition, the result of structure analysis was accordant with the result of FCA analysis.

## 4 Discussion

Genetic diversity parameters detected in the present study were similar or slightly higher than previous studies. Using 18 microsatellite loci in nine native Turkish sheep breeds, Yilmaz et al. (2014) observed mean number of alleles and effective alleles as 10.11 and 9.62, respectively, with AR ranging from 6.48 (OARFCB20) to 13.75 (OarFCB304). Yilmaz et al. (2015) reported mean number of alleles and effective alleles as 12.29 and 7.04, respectively in Gökçeada, Kıvırcık, Karacabey Merino, and Sakız sheep breeds by using 17 microsatellite loci, while Oner et al. (2014) reported mean number of alleles and effective alleles as 11.89 and 5.65, respectively in Kıvırcık, Pırlak, and Karacabey Merino sheep breeds by using 10 microsatellite loci. These results show that four native Turkish sheep populations hold high genetic diversity. The high mean PIC value (0.84) detected across 21 microsatellite loci indicates that these microsatellite loci have ability to show genetic diversity in native Turkish sheep populations. Although the mean PIC value was similar to previous studies conducted in native Turkish sheep breeds (Yilmaz et al., 2014, 2015), a lower PIC value was reported by Oner et al. (2014, 0.78), which used 10 microsatellite loci. Mean number of alleles and observed heterozygosity were reported as 8.78–0.78 and 8.33–0.74 in NRD and KRK, respectively (Yilmaz et al., 2014).

Although these values were higher than values detected in the present study, similar observed heterozygosity values were reported in some native Turkish sheep breeds including Karayaka (0.69), Morkaraman (0.60), Sakız (0.59), and Tuj (0.62) (Yilmaz et al., 2014). Additionally, similar observed heterozygosity values (ranging from 0.65 to 0.70) were reported in some native Italian sheep breeds (Ceccobelli at al., 2015, 2016). Lower $H_{E}$ (expected heterozygosity) values were reported in Akkaraman (0.73), Hemşin (0.69), Karayaka (0.72), Morkaraman (0.72), and Tuj (0.73) sheep breeds (Gutiérrez-Gil et al., 2006). Genetic diversity parameters detected in four populations show that native Turkish sheep populations still conserve enough genetic diversity.

In this study, expected heterozygosity values were higher than observed heterozygosity values, which may be due to presence of more homozygous individuals. Additionally, positive inbreeding coefficient values were detected due to presence of more homozygous individuals than heterozygous ones. Although similar $F_{\mathrm{IS}}$ values were reported in NRD (0.161) and KRK (0.131) sheep breeds (Yilmaz et al., 2014), lower $F_{\mathrm{IS}}$ values were reported in Akkaraman (Soysal et al., 2005; Gutiérrez-Gil et al., 2006) and some native Italian sheep breeds (Lasagna et al., 2011; Ceccobelli et al., 2016). It is not surprising that higher $F_{\mathrm{IS}}$ values were detected in KNG, since KNG was derived from the Akkaraman breed by selection studies in the past. A selection process focusing only on desired alleles for many traits such as meat and/or milk yield could cause increased homozygosity and inbreeding in a population. High inbreeding detected in GKR may be due to a decrease in the effective population size. Indeed, GKR was subjected to a conservation programme at Bahri Dagdas International Agricultural Research Institute authorized due to danger of extinction (Ertugrul et al., 2009). In addition, high but not dangerous levels of inbreeding were observed in NRD and KRK populations. Presence of null alleles or nonrandom mating in populations may cause increased $F_{\mathrm{IS}}$ values. It is reported that null allele frequency higher than 0.20 may affect estimates of some parameters such as observed heterozygosity and inbreeding coefficient (Mahammi et al., 2016; Chybicki and Burczyk, 2009). High null allele frequency (>0.2) was detected in only OarJMP29 and ETH10 loci. This could be attributed to breeding system and small population size rather than the null alleles.

All pairwise $F_{\mathrm{ST}}$ values were significant (p<0.01), implying that the four sheep populations could be considered genetically different. It has been highlighted in previous studies that the four sheep populations are varieties or types of the Akkaraman breed (Karaca et al., 2003; Karsli et al., 2011). However, presence of high genetic differentiation among the four sheep populations is not surprising considering that they are raised in different parts of Turkey under different climatic conditions along side their breeding practices (for instance, KNG sheep have been derived from Akkaraman via selection). Indeed, adaptation and in particular selection may change genotypic structure by affecting gene and genotype frequencies. On the contrary, detection of the highest pairwise $F_{\mathrm{ST}}$ value between NRD and KRK is surprising, because they are raised in the same part of Turkey. Therefore, investigation of maternal and paternal origins based on mtDNA and Y chromosome is needed.

In this study, the number of private alleles ranging from 7 (KRK) to 12 (KNG) was higher than previous studies conducted on native Turkish sheep breeds (Yilmaz et al., 2014, 2015). Number of private alleles is affected by number of migrated individuals across populations. It is normal to observe a low number of private alleles in the case of the presence of migrated individuals from different populations or populations coming from a common origin. The number of private alleles and pairwise $F_{\mathrm{ST}}$ values obtained in this study weaken the idea that these populations come from the same common ancestor (Akkaraman). However, it should not be neglected that adaptation and breeding processes of these populations for a long time may also lead to genetic differentiation. Compared to other populations, presence of the highest number of private alleles in the KNG population may be due to selection studies in this population. According to FCA analysis, Yilmaz et al. (2014) reported a higher genetic admixture among six Turkish native sheep populations including NRD and KRK. On the contrary, in the present study both Structure and FCA analysis revealed that NRD population clearly clustered separate from other studied populations and no admixture was detected between NRD and other sheep populations in FCA analysis. Moreover, GKR population also clustered separately based on Structure and FCA analysis.

## 5 Conclusions

This study revealed that KNG, GKR, NRD, and KRK populations still hold enough genetic diversity despite decreasing in population sizes in the last few decades. Inbreeding coefficient values were not at high levels affecting the sustainable use of NRD and KRK populations in the future. Nevertheless, high inbreeding coefficients were detected in KNG and GKR, which requires taking measures in order to decrease inbreeding. In this study, genetic differentiation coefficients and phylogenetic analysis show that GKR and NRD populations, which have been accepted as Akkaraman varieties, have became genetically different from the Akkaraman breed. In order to clarify the genetic differentiation of GKR and NRD from the Akkaraman breed, more comprehensive molecular studies (such as SNP chips or next-generation sequencing etc.) are needed.

## Data Availability

The data sets are available upon request from the corresponding author.
